# Neuroprotective Effects of Cerebral Ischemic Preconditioning in a Rat Middle Cerebral Artery Occlusion Model: The Role of the Notch Signaling Pathway

**DOI:** 10.1155/2018/8168720

**Published:** 2018-08-06

**Authors:** Li Chen, Kuan Huang, Rong Wang, Qiong Jiang, Zhenghua Wu, Weidong Liang, Rui Guo, Lifeng Wang

**Affiliations:** ^1^First Affiliated Hospital of Gannan Medical University, Ganzhou, Jiangxi 341000, China; ^2^Gannan Medical University, Ganzhou, Jiangxi 341000, China

## Abstract

Cerebral ischemia-reperfusion (I/R) injury is a major problem worldwide. The Notch signaling pathway plays an important role in neural progenitor cell differentiation and in the inflammatory response after central nervous system injury. This study evaluated whether the neuroprotective effect of cerebral ischemic preconditioning (cIPC) is mediated by the preactivation of the Notch signaling pathway. A rat middle cerebral artery occlusion/reperfusion (MCAO/R) model and glucose deprivation/reoxygenation (OGD/R) cell model were constructed to detect the neuroprotective effects of cIPC. In in vivo experiments, cIPC reduces the neurological functional deficit, cerebral infarction, and cellular apoptosis in the hippocampus induced by middle cerebral artery occlusion/reperfusion (MCAO/R), thus indicating that cIPC can improve neurologic function. Moreover, cIPC can reveal the expression peak of Jagged1, Notch1, NICD, and Hes1 protein, thereby indicating that cIPC can preactivate Notch signaling. However, cIPC-induced improvements in neurologic function are compromised by the *γ*-secretase inhibitor N-(N-(3,5-difluorophenacetyl)-1-alanyl)-S-phenylglycine t-butyl ester (DAPT). In in vitro experiments, OGD preconditioning (OGDPC) can clearly upregulate Notch1 expression in the OGD/R-treated neuron and neural stem cell. Notch1 pre-overexpression can decrease neuron death and apoptosis under OGD/R treatment. Notch1 pre-overexpression can decrease the percentage of G1 stage cells and increase the percentage of S stage cells in OGD/R-treated neural stem cell. Furthermore, Notch1 pre-knockdown has the opposite effect on cell survival, apoptosis, and cycle in both OGD/R-treated neuron and neural stem cell. In conclusion, our results demonstrate that the neuroprotective effects of cIPC in a rat MCAO/R model are mediated by the preactivation of the Notch signaling pathway.

## 1. Introduction

Cerebral ischemia-reperfusion (I/R) injury is a major problem worldwide. Cerebral I/R injury often causes irreversible brain damage that leads to functional impairment and/or neuronal death [1, 2]. The treatment for this disorder remains palliative only. Currently, this illness has no effective pharmacotherapy. Ischemic preconditioning (IPC), elicited by a nonfatal brief occlusion of blood flow, is a technique for producing resistance to the loss of blood supply. Emerging data from clinical trials have shown that IPC is a therapeutic strategy against subsequent fatal ischemic insults in vital organs (e.g., kidney and heart) [3–7]. However, cerebral IPC (cIPC), a transient sublethal cerebral ischemia, cannot be applied in clinic because of security and ethical concerns. Therefore, elucidating the regulating mechanism of cIPC is important to discover drugs that behave in a similar way.

Notch signaling is evolutionarily conserved from Drosophila to humans. It plays an important role in neural stem cell maintenance and neurogenesis in the embryonic brain and adult brain [8, 9]. During neocortical development, Notch signaling inhibits neuronal differentiation and maintains the neural stem/progenitor cell pool to enable successive waves of neurogenesis, which are followed by gliogenesis [9]. Aside from its role in neural development, Notch signaling also has a role in cerebral I/R injury [3, 10]. The Notch signaling pathway is important in neural progenitor cell differentiation and in the inflammatory response after central nervous system injury [11]. A recent study showed that the neuroprotective effects of isoflurane preconditioning in a murine transient global cerebral I/R model are mediated by the preactivation of the Notch signaling pathway [12, 13]. However, whether the Notch signaling pathway plays a role in the neuroprotective effects of cIPC remains unclear.

No clinical data are available on the neuroprotective effect of cIPC. Therefore, the function and the regulating mechanism of cIPC are only studied in animal models, such as the middle cerebral artery occlusion (MCAO) model. In this study, we investigated the relationship between changes in the Notch signaling pathway and the neuroprotection induced by cIPC in vivo and in vitro. Our hypothesis is that the neuroprotection of cIPC is mediated by the preactivation of the Notch signaling pathway.

## 2. Materials and Methods

### 2.1. Experimental Animals

Ninety male Sprague–Dawley (SD) rats (200–220 g, 9 weeks old) were obtained from Vital River Laboratories (Beijing, China). The animals were allowed free access to food and water under controlled room temperature (25°C) and lighting (14:10 light/dark cycle) conditions for this study. All animal experimental procedures were approved by the Institute of Animal Care and Use Committee of First Affiliated Hospital of Gannan Medical College and performed in accordance with the Guide for the Care and Use of Laboratory Animals (NIH publication no. 85-23, National Academy Press, Washington, DC, USA, revised 1996).

### 2.2. MCAO/Reperfusion (MCAO/R) and cIPC Model

The MCAO model was performed as described previously [14], with minor modifications. Briefly, the rats were placed in the supine position, with the limbs taped to the operating table, after they were anesthetized with an intraperitoneal injection of 350 mg/kg chloral hydrate. After a midline skin incision, the left external carotid artery was exposed, and its branches were ligated. A nylon monofilament (diameter = 0.26 mm) coated with silicon was introduced into the internal carotid artery through the common carotid artery and advanced until faint resistance was felt. After 90 min of occlusion, blood flow was restored by withdrawing the nylon thread to allow reperfusion. Sham-operated control rats received the same procedure except filament insertion. The cIPC operation was the same as the MCAO, but the occlusion was conducted for 10 min. The rectal temperature was maintained at 37 ± 0.5°C by lamp irradiation. All surgical procedures were performed under sterile conditions.

### 2.3. Experimental Groups

Ninety rats were randomly allocated to six groups: sham group: a healthy control group that underwent sham surgery; cIPC group: an MCAO/R model group that received 10 min of ischemia and reperfusion for one day; MCAO/R group: an MCAO model group that received 90 min of ischemia and reperfusion for 2 h for one or seven days; cIPC+ MCAO/R group: rats initially received 10 min of ischemia after one day of reperfusion and then received 90 min of ischemia and reperfusion for 2 h for one or seven days; N-[N-(3,5-difluorophenacetyl)-l-alanyl]-S-phenylglycine tert-butyl ester (DAPT) + cIPC + MCAO/R: 100 mg/kg *γ*-secretase inhibitor N-(N-(3,5-difluorophenacetyl)-1-alanyl)-S-phenylglycine t-butyl ester (DAPT) was injected intraperitoneally 3 h before cIPC+ MCAO/R surgery; and DMSO+ cIPC + MCAO/R group: equal volume vehicle DMSO was injected intraperitoneal injection 3 h before cIPC+ MCAO/R surgery.

### 2.4. Neurological Function Testing

Neurological functional scores were assessed 24 h postoperatively using Longa's method [14]. Neurological status was scored on a four-point scale as follows: 0 = no neurological symptoms; 1 point = inability to completely extend the right front paw; 2 = rotating while crawling and falling to the right side; 3 = inability to walk without assistance; and 4 = unconscious. Rats scoring 1–3 points met the inclusion criteria for the experiment, and those scoring 0 or 4 were excluded.

### 2.5. Measurement of the Cerebral Infarction Volume

After neurological function testing, the rats were killed for infarction volume analysis. Whole brains were quickly removed and frozen at 20°C for 30 min. The brains were cut into five coronal sections. The first cut was at the midpoint between the anterior pole and the optic chiasm. The slices were stained in 0.5% triphenyltetrazolium chloride (TTC) solution at a temperature of 37°C for 15 min away from light and then fixed with 4% paraformaldehyde for 4 h. A digital camera and an image analysis system were used for the image acquisition and analysis. The infarct size was determined using Image Pro-Plus 6.0 software (Media Cybernetics, Silver Springs, MD, USA). Infarct ratio (%) = the calibrated infarct volume/volume of the contralateral hemisphere.

### 2.6. Terminal Deoxynucleotidyl Transferase-Mediated 2′-Deoxyuridine 5′-Triphosphate Nick-End Labeling (TUNEL) Assay for Apoptotic Nuclei

For each rat, six sections were analyzed using the In Situ Cell Death Detection Kit, AP (Roche Molecular Biochemicals, Mannheim, Germany) according to the manufacturer's protocols. For image analysis, three random fields (200×) from each rat were observed for TUNEL-positive cells using fluorescence microscopy (Carl Zeiss, Jena, Germany).

### 2.7. Neuron and Neural Stem Cell (NSC) Isolation

Hippocampal neuron was isolated from specific pathogen-free grade fetal SD rats (14 days, Vital River Laboratories) and cultured as described previously [15]. The isolated neuron was identified by detecting the expression of MAP2 and GFAP using immunofluorescence as described previously [16]. NSC was isolated from specific pathogen-free grade fetal SD rats (14 days, Vital River Laboratories) as described previously [16]. Isolated NSC was identified by detecting the expression of nestin and BrdU using immunofluorescence as described previously [16].

### 2.8. Notch1 Overexpression and Knockdown by Adenovirus-Mediated Transduction

The shRNA sequence targeting GCAAUAAGGUCUGCAACCU and the full open reading frame of Notch1 were cloned into a linearized adenovirus plasmid GV314 (Genechem) with T4 DNA ligase and transfected into competent* Escherichia coli* cells. Positive clones were selected by ampicillin resistance and then sequenced. The Notch1 overexpression adenovirus (Ad-Notch1) and Notch1 shRNA adenovirus (Ad-sh-Notch1) were packaged in HEK293T cells and purified with an Adeno-X™ Virus Purification Kit (BD Biosciences, San Jose, CA, USA). The endpoint dilution method was used to determine the viral titer. Adenovirus particles expressing a scrambled sequence (Ad-NC; purchased from Genechem) served as the negative control. For cell infection, the total NSC and neuron were seeded in six-well plates with or without coverslips. After 24 h, the virus was added to the cells at a multiplicity of infection of 100 in the presence of 4 *μ*g/ml polybrene. Cells not transfected with the virus were classified as MOCK. Twenty-four hours after infection, the medium was changed to complete DMEM and then changed every three days. After 72 h, the viral infection efficiency was assessed under fluorescence microscopy. Cells were harvested for experiments on day 8 after infection.

### 2.9. Cell Model and Experimental Group

Sham: cells were cultured in a complete medium at 37°C in a humidified 5% CO_2_ incubator. OGD/R injury model: cells were cultured in a sugar-free and serum-free culture medium in a Billups–Rothenberg chamber with 94% N_2_, 1% O_2_, and 5% CO_2_ at 37°C for 2 h and then cultured in a complete medium at 37°C in a humidified 5% CO_2_ incubator for 24 h. OGDPC model: cells were cultured in a sugar-free and serum-free culture medium in a Billups–Rothenberg chamber with 94% N_2_, 1% O_2_, and 5% CO_2_ at 37°C for 20 min and then cultured in a complete medium at 37°C in a humidified 5% CO_2_ incubator for 20 min. OGDPC+ OGD/R model: cells were cultured in the same condition as in the OGDPC model and then cultured in the same condition as in the OGD/R model.

### 2.10. Western Blotting

The total protein of the hippocampal tissue or cells was isolated using lysis buffer containing protease inhibitors, and the protein concentration of each sample was estimated by the Lowry method using a protein assay kit. Aliquots of lysates containing 20 lg of protein were separated by electrophoresis on 8% gradient sodium dodecyl sulfate-polyacrylamide gels (SDS-PAGE) and transferred to nitrocellulose membranes (Bio-Rad Laboratories, Richmond, CA, USA). After blocking for 1 h at room temperature with 5% (w/v) skim milk powder, blots were incubated overnight at 4°C with the primary antibodies anti-Jagged1, anti-Notch1, anti-NICD, and anti-Hes1 (Abcam, Cambridge, MA, USA). Anti-GAPDH antibody was used as the internal control. Anti-rabbit antibody was used as the secondary antibody, and antigens were detected using the standard chemiluminescence method (ECL; Millipore Biotech, Billerica, MA, USA).

### 2.11. Cell Proliferation Assays

Cell proliferation was measured using the CellTiter 96 AQueous One Solution Cell Proliferation Assay Kit (Promega, Madison, WI, USA) according to the manufacturer's protocol. Adenovirus-transduction NSC and neuron cells (1 × 10^4^) were seeded onto a 96-well plate. The cells were exposed to a hypoxia condition and cultured in a Billups–Rothenberg chamber with 94% N_2_, 1% O_2_, and 5% CO_2_ at 37°C for 2 h. Subsequently, the cells were maintained at 37°C in a humidified 5% CO_2_ incubator for another 24, 48, or 72 h. After culturing, 10 *μ*L of the CellTiter 96 AQueous One Solution reagent was added to each well. The cells were then incubated for 4 h at 37°C. Absorbance was measured at 490 nm using a microplate reader (Multiskan MK3, Thermo Scientific, Vantaa, Finland). The survival rate was calculated using the following formula: survival rate = (OD_test_/OD_negative  control_) × 100%.

### 2.12. Flow Cytometry Analysis

Adenovirus-transduction NSC and neuron cells (2 × 10^5^) cells were seeded onto a 24-well plate. The cells were exposed to a hypoxia condition, cultured in a Billups–Rothenberg chamber with 94% N_2_, 1% O_2_, and 5% CO_2_ at 37°C for 2 h. Subsequently, the cells were maintained at 37°C in a humidified 5% CO_2_ incubator for reperfusion. After reperfusion for 24 h, the cells were harvested for cell apoptosis and cell cycle analysis. Annexin V-FITC apoptosis detection and cell cycle detection kits were used to analyze the apoptosis rate and the cell cycle distribution according to the manufacturer's protocols (Keygen, Nanjing, China). The percentage of apoptotic cells and the cell cycle distribution were analyzed by flow cytometry (BD Biosciences, San Jose, CA, USA). Each experiment was repeated three times.

### 2.13. Statistical Analysis

All data were expressed as mean±SD. Statistical analyses were performed using the Statistical Package for the Social Sciences (SPSS) v19.0 software (SPSS Inc., Chicago, IL, USA). We compared the groups using one-way ANOVA, followed by post hoc tests of least significant difference (LSD) for multiple pairwise comparisons. A value of *p* < 0.05 was considered statistically significant.

## 3. Results

### 3.1. cIPC Reduced the Neurological Functional Deficit and Cerebral Infarction Induced by MCAO/R

**Figure 1:**
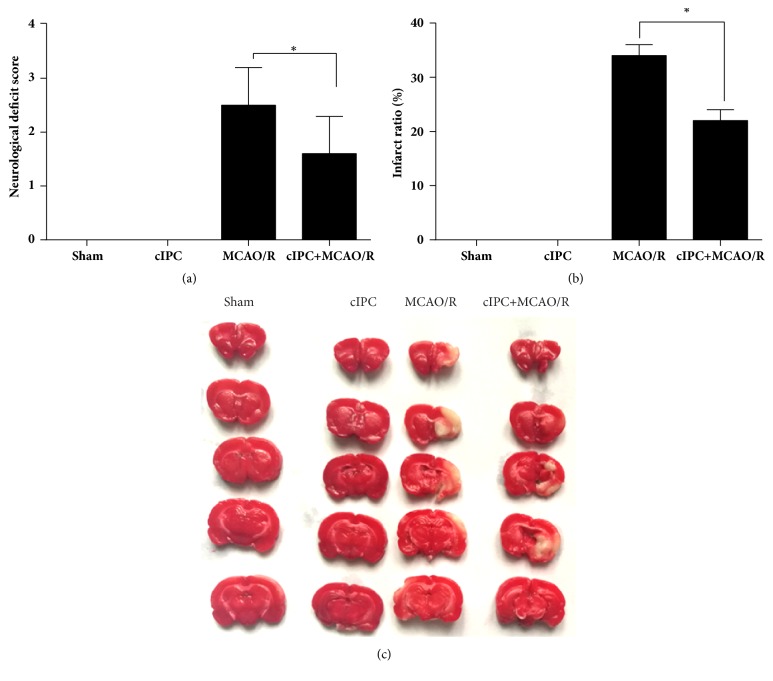
cIPC reduced the neurological functional deficit and cerebral infarction induced by MCAO/R for 24 h. (a) The neurological functional deficit score was examined to evaluate the effect of cIPC on the neurological functional deficit induced by MCAO/R. (b) TTC staining was conducted to evaluate the effect of cIPC on cerebral infarction induced by MCAO/R. (a) is the infarct ratio calculated according to the results of TTC staining. (c) is the represented image of TTC staining. ^*∗*^*p* < 0.05.

After reperfusion for one day, neurological function testing was performed to examine the neurological deficit score of each rat group. The results showed that cIPC had no obvious effect on the neurological deficit score unlike in the sham group (Figure 1(a)). The neurological deficit score of the MCAO/R group was significantly higher than that of the sham group (Figure 1(a)). However, this increase could be decreased by cIPC (Figure 1(a)). The results of TTC staining showed no cerebral infarction in both sham and cIPC groups (Figures 1(b) and 1(c)). However, the infarct ratio of the MCAO/R group was significantly higher than that of the sham group, and this increase could be decreased by cIPC (Figures 1(b) and 1(c)).

### 3.2. cIPC Reduced Cellular Apoptosis in the Hippocampus Induced by MCAO/R

**Figure 2:**
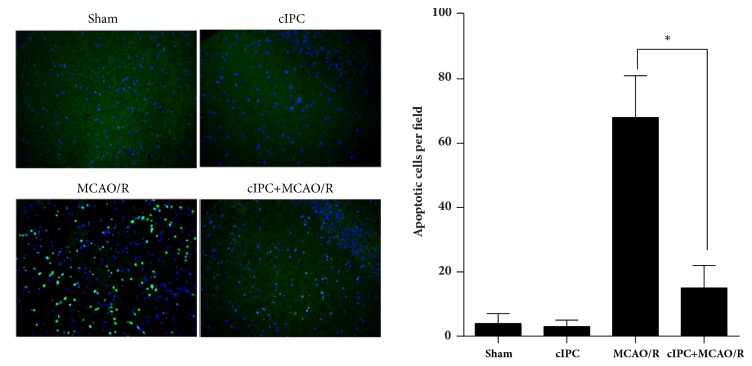
cIPC reduced cell apoptosis in the hippocampus induced by MCAO/R for 24 h. TUNEL assay was conducted to evaluate the effect of cIPC on the hippocampus cell apoptosis induced by MCAO/R. On the left is the represented image of the TUNEL assay (200×). The blue color is cell nucleus stained by DAPI. Green color is TUNEL-positive cells. On the right is the statistical result of the number of apoptotic cells. ^*∗*^*p* < 0.05.

After reperfusion for one day, the total brains of each rat group were obtained for TUNEL assay for apoptotic nuclei. The apoptotic nuclei in the hippocampus were analyzed. As shown in Figure 2, no obvious difference was found in the number of apoptotic nuclei in the hippocampus of rats in the sham and cIPC groups, thus indicating that cIPC could not increase the number of apoptotic cells. The number of apoptotic nuclei in the hippocampus of rats in the MCAO/R group significantly increases compared with that in the sham group, and this increase could be decreased by cIPC.

### 3.3. Effect of cIPC on the MCAO/R-Induced Notch Signaling Activation

**Figure 3:**
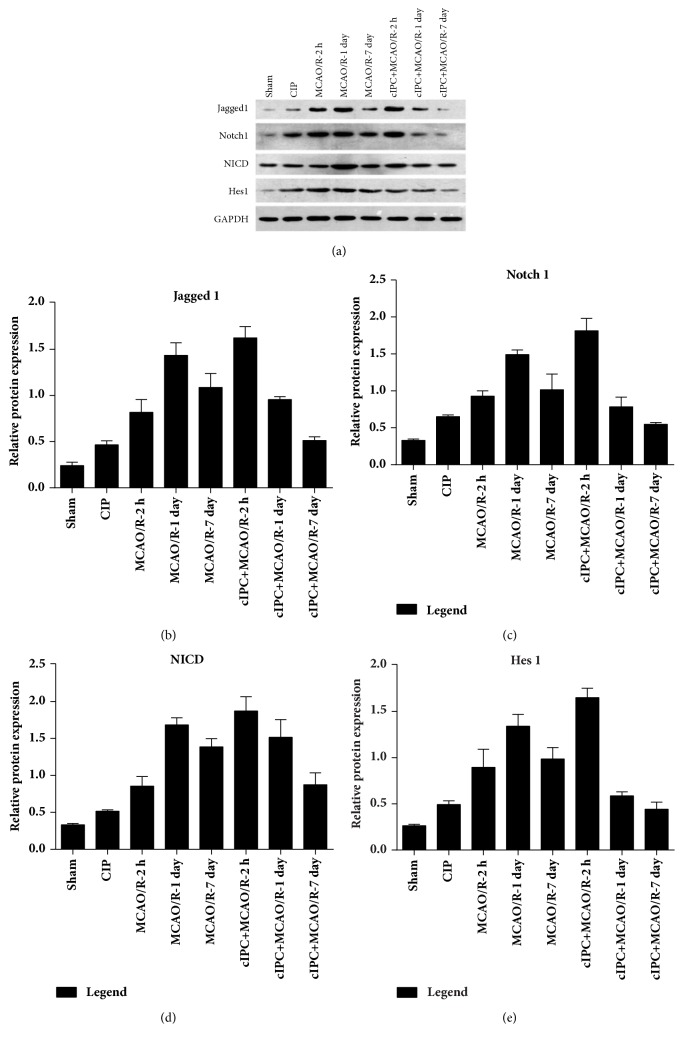
cIPC could advance the expression peak of Jagged1, Notch1, NICD, and Hes1 protein in the hippocampus of the rat (MCAO/R) model. (a) The represented images. (b–e) Relative protein level of Jagged 1, Notch1, NICD, and Hes1 expressed as the relative ratio of density of the target protein to the reference protein GAPDH.

To investigate the possible mechanism of the neuroprotective effects of cIPC, we detected the expression levels of Jagged1, Notch1, NICD, and Hes1, which belong to the Notch signaling pathway, in the hippocampus. Western blot analyses showed that cIPC clearly increased the expressions of Jagged1, Notch1, NICD, and Hes1 protein unlike in the sham groups (Figures 3(a), 3(b), 3(c), 3(d), and 3(e)). MCAO/R also increased the expressions of Jagged1, Notch1, NICD, and Hes1 protein unlike in the sham group 2 h, 1 day, and 7 days after reperfusion, and the peak of these increases occurred one day after reperfusion (Figures 3(a), 3(b), 3(c), 3(d), and 3(e)). At the same time point, the expressions of Jagged1, Notch1, NICD, and Hes1 protein in the cIPC+MCAO/R group were higher than those in the MCAO/R group, thus indicating that cIPC could advance the expression peaks of Jagged1, Notch1, NICD, and Hes1 protein (Figures 3(a), 3(b), 3(c), 3(d), and 3(e)). All these results indicated that cIPC could preactivate the Notch signaling.

### 3.4. cIPC-Induced Improvements in Neurologic Function Are Compromised by *γ*-Secretase Inhibitor DAPT

**Figure 4:**
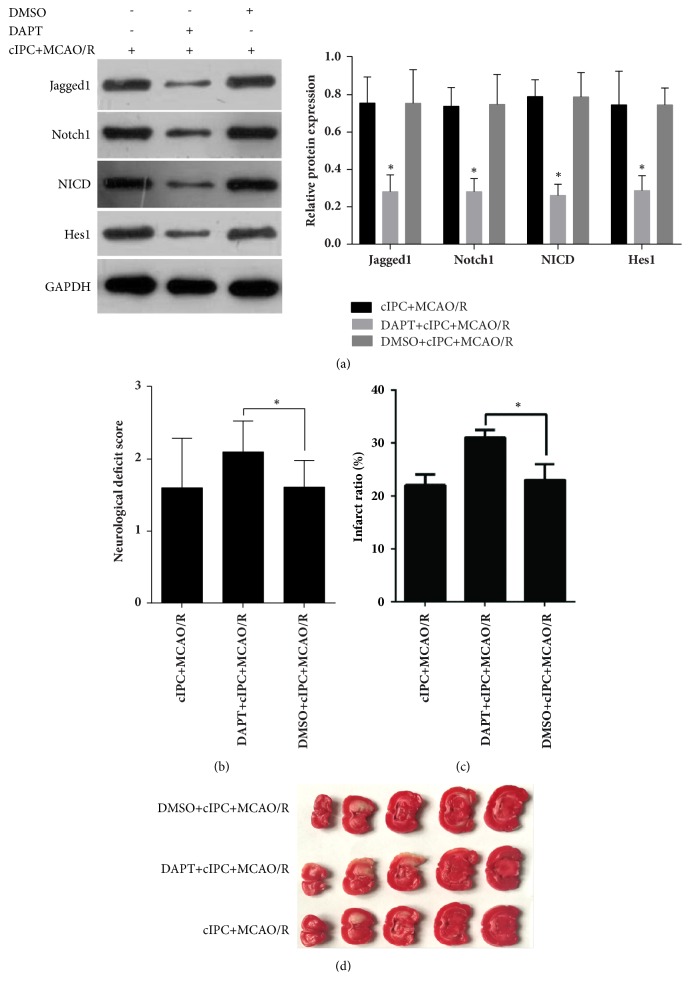
The cIPC-induced improvements in the neurological functional deficit and cerebral infarction induced by MCAO/R are compromised by the *γ*-secretase inhibitor DAPT. (a) Jagged1, Notch1, NICD, and Hes1 expression levels in the brain tissues of DAPT-treated rats. (b) The neurological functional deficit score was examined to evaluate the effect of DAPT and/or cIPC on the neurological functional deficit induced by MCAO/R. (c-d) TTC staining was conducted to evaluate the effect of DAPT and/or cIPC on cerebral infarction induced by MCAO/R. The top figure is the infarct ratio calculated according to the results of TTC staining (c). The bottom photo is the represented image of TTC staining (d). ^*∗*^*p* < 0.05.

**Figure 5:**
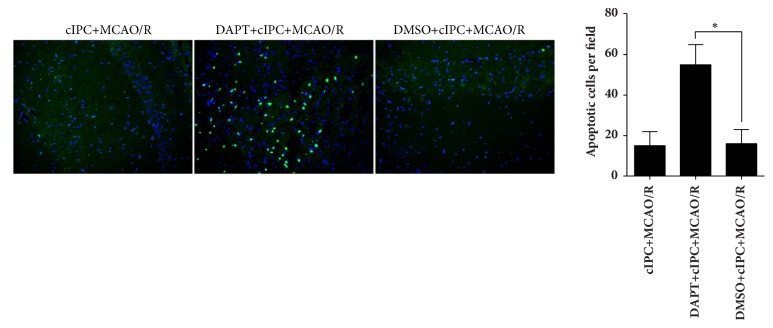
cIPC reduced cell apoptosis in the hippocampus induced by MCAO/R for 24 h. TUNEL assay was conducted to evaluate the effect of DAPT and/or cIPC on the hippocampus cell apoptosis induced by MCAO/R. The photo on the left is the represented image of TUNEL assay (200×). The figure on the right is the statistical result of the number of apoptotic cells. ^*∗*^*p* < 0.05.

To further determine the role of Notch signaling in the cIPC-induced neuroprotective effects in the rat middle MCAO/R model, *γ*-secretase inhibitor DAPT or vehicle DMSO was injected intraperitoneally 3 h before cIPC. The results of Western blot showed that Jagged1, Notch1, NICD, and Hes1 expression levels were decreased in the brain tissues of DAPT-treated rats (Figure 4(a)), indicating that Notch signaling was successfully blocked by DAPT pretreatment. The results of the neurological function testing showed that DAPT pretreatment could increase the neurological deficit score, which was decreased by cIPC in the rat MCAO/R model (Figure 4(b)). In addition, the results of TTC staining showed that DAPT pretreatment could increase the infarct ratio, which was decreased by cIPC in the rat middle MCAO/R model (Figures 4(c) and 4(d)). DAPT pretreatment could also increase the number of apoptotic nuclei in the hippocampus decreased by cIPC in the rat middle MCAO/R model (Figure 5).

### 3.5. Effect of OGDPC on Notch1 Expression in OGD/R-Treated Neuron and NSC

**Figure 6:**
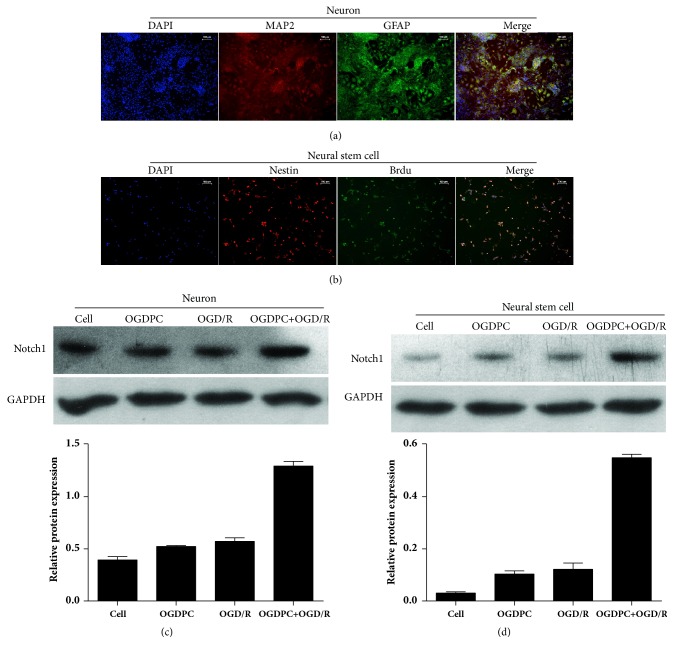
Effect of OGDPC on Notch1 expression in OGD/R-treated neuron and neural stem cell. (a) The isolated neuron was identified by detecting the expression of MAP2 and GFAP using immunofluorescence. (b) Isolated NSC was identified by detecting the expression of nestin and BrdU using immunofluorescence. (c-d) Expression levels of Notch1 in the neuron (c) or neural stem cell (d) models of sham, OGDPC, OGD/R, and OGDPC + OGD/R.

MAP2 and GFAP were highly expressed in the isolated hippocampal neuron (Figure 6(a)). Nestin and BrdU were highly expressed in isolated NSC (Figure 6(b)). These results indicated that the hippocampal neuron and NSC were successfully isolated and could be used for the subsequent assays. Then, the cell models of sham, OGDPC, OGD/R, and OGDPC + OGD/R were constructed. The Western blotting results showed that OGDPC and/or OGD/R treatment could upregulate the protein expression of Notch1 in both neuron and neural stem cell (Figures 6(c) and 6(d)). In addition, the Notch1 expression induced by OGDPC + OGD/R was higher than that induced by OGD/R only at the same time point after reperfusion, thereby indicating that OGDPC could preactivate Notch signaling.

### 3.6. Effect of Notch1 Overexpression and Silencing on OGD/R-Treated Neuron Cell Survival and Cellular Apoptosis

**Figure 7:**
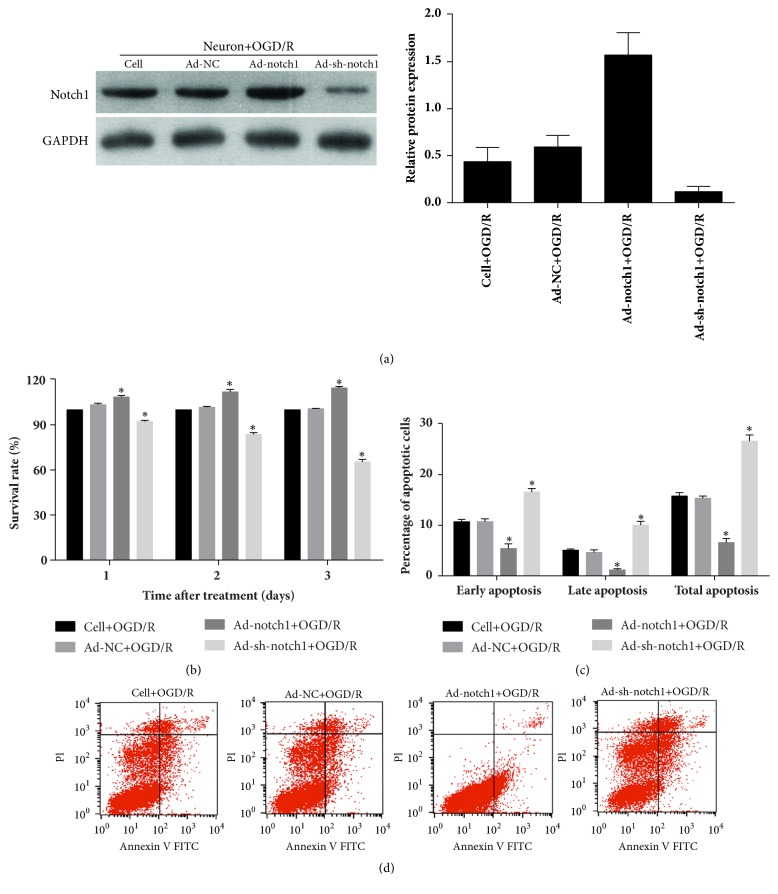
Effect of Notch1 overexpression and silencing on OGD/R-treated neuron cell survival and cellular apoptosis. (a) Notch1 expression in neuron was overexpressed or silenced by transfected Notch1 overexpression adenovirus (Ad-Notch1) and Notch1 shRNA adenovirus (Ad-sh-Notch1). (b) The survival rate of each cell group was calculated by detecting the absorbance at 490 nm using the CellTiter 96 AQueous One Solution Cell Proliferation Assay Kit. (c-d) The percentage of apoptotic cells of each cell group was detected by flow cytometry analysis. (c) Statistical result of the percentage of apoptotic cells. (d) Represented images. ^*∗*^*p* < 0.05.

After transfection with Ad-NC, Ad-Notch1, and Ad-sh-Notch1 for three days, the expression level of Notch1 in neuron was detected using Western blotting. The results showed that Notch1 was successfully knockdown after transfected with Ad-sh-Notch1 and successfully overexpressed after transfection with Ad-Notch1 (Figure 7(a)). Then, the effect of Notch1 overexpression and silencing on OGD/R-treated neuron cell survival and cellular apoptosis was detected using cell proliferation assays and flow cytometry analysis. The results showed that the survival rate of a neuron transfected with Ad-Notch1 was higher than that transfected with Ad-NC one, two, and three days after OGD/R treatment (Figure 7(b)). The percentage of apoptotic neuron transfected with Ad-Notch1 was significantly lower than that transfected with Ad-NC 2 h after OGD/R treatment (Figures 7(c) and 7(d)). These results indicated that Notch1 pre-overexpression could decrease neuron death and apoptosis under OGD/R treatment. In addition, Notch1 pre-knockdown could advance neuron death and apoptosis under OGD/R treatment (Figures 7(b), 7(c), and 7(d)).

### 3.7. Effect of Notch1 Overexpression and Silencing on the OGD/R-Treated NSC Cell Cycle

**Figure 8:**
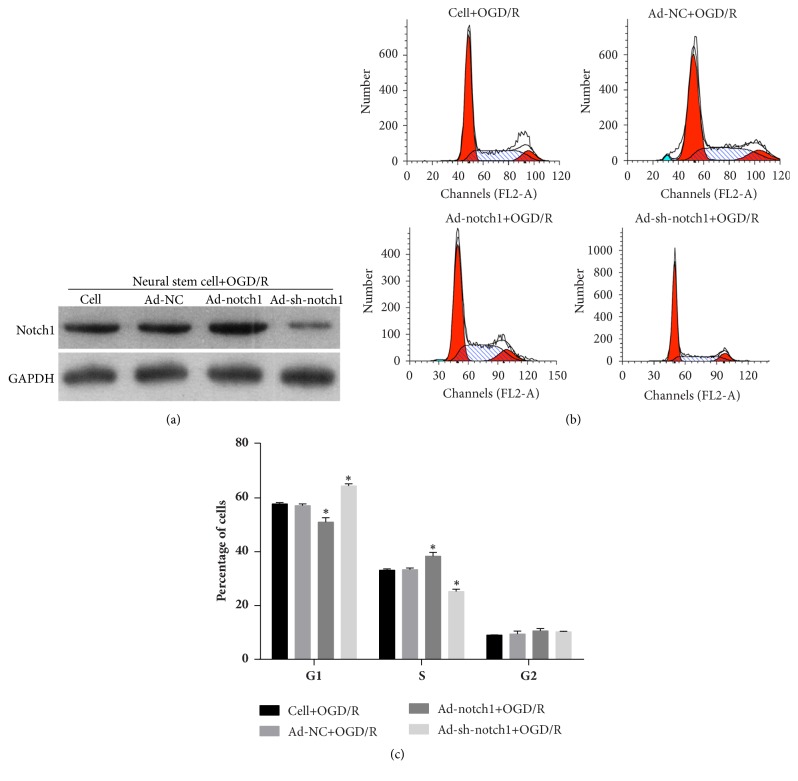
Effect of Notch1 overexpression and silencing on OGD/R-treated neural stem cell cycle. (a) The Notch1 expression in OGD/R-treated neural stem cell was overexpressed or silenced by transfected Notch1 overexpression adenovirus (Ad-Notch1) and Notch1 shRNA adenovirus (Ad-sh-Notch1). (b-c) The percentage of G1, S, and G2 stage cells of each cell group cell was detected by flow cytometry analysis. (b) Represented images. (c) Statistical result of the percentages of G1, S, and G2 stage cells. ^*∗*^*p* < 0.05.

After transfection with Ad-NC, Ad-Notch1, and Ad-sh-Notch1 for three days, the expression level of Notch1 in neural stem cells was detected using Western blotting. The results showed that Notch1 was successfully knocked down after transfection with Ad-sh-Notch1 and successfully overexpressed after transfection with Ad-Notch1 (Figure 8(a)). Then, the effect of Notch1 overexpression and silencing on OGD/R-treated neuron cell cycle was detected using flow cytometry analysis. The results showed that the percentage of G1 stage cells decreased after transfection with Ad-Notch1 and increased after transfection with Ad-sh-Notch1 (Figures 8(b) and 8(c)). In addition, the percentage of S stage cells increased after transfection with Ad-Notch1 and decreased after transfection with Ad-sh-Notch1 (Figures 8(b) and 8(c)). These results indicated that Notch1 pre-overexpression could promote neuron stem cell division.

## 4. Discussion

Cerebral I/R injury induced by irreversible functional impairment and/or neuronal death is a controversial issue in the treatment of cerebral I/R injury-related diseases, such as stroke and cerebral trauma [1, 2]. Therefore, developing an effective therapy to reduce the harm of cerebral I/R is critical. cIPC is one potential treatment option, but it cannot be applied clinically because of security and ethical concerns. Further illuminating the therapeutic effect and the regulating mechanism of cIPC is important to discover drugs that behave in similar ways. The present study demonstrates that cIPC has a neuroprotective effect in the rat MCAO/R model. Moreover, cIPC induces the preactivation of the Notch signaling pathway, and the neuroprotection induced by cIPC is reduced by the administration of the Notch signaling inhibitor DAPT prior to cIPC. Our study provides theoretical basis for the molecular mechanism of cIPC and for the targeted therapy of cerebral I/R injury-related diseases.

Our result shows that cIPC significantly inhibits cerebral infarction and cellular apoptosis and improves the neurological functional deficit, in the hippocampus induced by MCAO/R. This result indicates that cIPC has a neuroprotective effect in the rat MCAO/R model. The MCAO model is a reliable method for studying reversible regional ischemia [14]. Therefore, our model demonstrates that cIPC has a neuroprotective on lethal ischemic insult. This result is consistent with those of previous studies, and it indicates that our model can be used to examine the mechanism of cIPC.

Although significant progress has been made toward identifying some of the major molecules involved in the neuroprotection of cIPC, the mechanisms behind the formation of cIPC protection remain largely unknown [17]. Notch signaling plays a key role in brain physiology and pathology [8, 18, 19]. However, its role in the formation of cIPC protection remains unclear. Our results in vivo and in vitro will fill the current biological gap. First, we found that cIPC could advance the expression peak of Jagged1, Notch1, NICD, and Hes1 protein in the cIPC+MCAO/R group unlike in the MCAO/R group. Jagged1 is the ligand for the receptor Notch1 [20]. Hes1 is a major transcriptional downstream regulator of the Notch signaling pathway [21]. Therefore, cIPC can certainly preactivate the Notch signaling. Second, we found that the cIPC-induced improvements in neurologic function are compromised by the *γ*-secretase inhibitor DAPT. Third, Notch1 pre-knockdown can advance primary neuron death and apoptosis under I/R treatment. Fourth, Notch1 pre-overexpression can promote neuron stem cell division under I/R treatment. These results reveal that Notch signaling is involved in the neuroprotective effects of cIPC in the rat MCAO/R model. However, our present result seems to contradict some previous public reports. There are many reports showing detrimental function of Notch signaling in ischemic stroke [19, 22–25]. It is sure that Notch1 signaling pathway can be activated by cerebral ischemia and hypoxia. However, the function of the downstream genes regulated by Notch1 signaling pathway and four prominent interacting pathways (NF-*κ*B, p53, HIF-1*α*, and Pin1) is complex [26]. So we predict that Notch1 signaling pathway may play different role in different physiological environment by regulated the expression of different downstream genes. Our predication may be partly supported by some previous studies. For example, the preactivation of the Notch signaling pathway contributes to the neuroprotective effects of isoflurane preconditioning in a murine transient global cerebral I/R model [12], and the activation of the canonical Notch signaling pathway is involved in the ischemic tolerance induced by sevoflurane preconditioning in a mouse MCAO/R model [27]. So the role of Notch1 signaling pathway in cerebral I/R injury-related diseases is still inconclusive and more study is needed to carry out.

Although Notch signaling is certain to be involved in cerebral I/R injury [10, 12, 18], no direct evidence demonstrates the role of Notch signaling in the neuroprotective effects of cIPC. Therefore, our results provide further evidence to confirm the role of Notch signaling in cerebral I/R injury and its possibility as a target for the treatment of cerebral I/R injury-related diseases. Nevertheless, more effort is needed to find the specific activator of the Notch signaling pathway to simulate the function of cIPC.

In conclusion, cIPC can activate and advance the peak expression of Notch signaling after MCAO/R. The neuroprotective effect of cIPC on cerebral I/R is diminished by blocking the Notch signal. These findings indicate that Notch signals are crucial pathways for inducing ischemic tolerance by cIPC. This work establishes a foundation for future research to investigate new mechanisms and therapeutic targets of cerebral I/R injury-related diseases.

## Data Availability

The data used to support the findings of this study are available from the corresponding author upon request.
